# Green Biosynthesis of Zinc Oxide Nanoparticles Using *Pluchea indica* Leaf Extract: Antimicrobial and Photocatalytic Activities

**DOI:** 10.3390/molecules28124679

**Published:** 2023-06-09

**Authors:** Abdulaziz A. Al-Askar, Amr H. Hashem, Nadeem I. Elhussieny, Ebrahim Saied

**Affiliations:** 1 Department of Botany and Microbiology, Faculty of Science, King Saud University, P.O. Box 2455, Riyadh 11451, Saudi Arabia; 2 Botany and Microbiology Department, Faculty of Science, Al-Azhar University, Cairo 11884, Egypt; 3 Department of Life Science and Chemistry, Constructor University, 28759 Bremen, Germany; 4 Institute of Environmental Biology and Biotechnology, University of Applied Sciences Bremen, Am Neustadtwall 30, 28199 Bremen, Germany

**Keywords:** green biosynthesis, zinc oxide nanoparticles, antimicrobial activity, photocatalytic activity

## Abstract

Nanotechnology is playing a critical role in several essential technologies with nanoscale structures (nanoparticles) in areas of the environment and biomedicine. In this work, the leaf extract of *Pluchea indica* was utilized to biosynthesize zinc oxide nanoparticles (ZnONPs) for the first time and evaluated for antimicrobial and photocatalytic activities. Different experimental methods were used to characterize the biosynthesized ZnONPs. The biosynthesized ZnONPs showed maximum Ultraviolet–visible spectroscopy (UV-vis) absorbance at a wavelength of 360 nm. The X-Ray diffraction (XRD) pattern of the ZnONPs exhibits seven strong reflection peaks, and the average particle size was 21.9 nm. Fourier-transform infrared spectroscopy (FT-IR) spectrum analysis reveals the presence of functional groups that help in biofabrication. The existence of Zn and O was confirmed by the Energy-dispersive X-ray (EDX) spectrum and the morphology by SEM images. Antimicrobial studies showed that the biosynthesized ZnONPs have antimicrobial efficacy against *Escherichia coli, Pseudomonas aeruginosa, Enterococcus faecalis, Bacillus subtilis, Staphylococcus aureus, Candida albicans* and *Cryptococcus neoformans* where inhibition zones at concentration 1000 µg/mL were 21.83 ± 0.76, 13.0 ± 1.1, 14.9 ± 0.85, 24.26 ± 1.1, 17.0 ± 1.0, 20.67 ± 0.57 and 19.0 ± 1.0 mm respectively. Under both dark and sunlight irradiation, the photocatalytic activity of ZnONPs was evaluated towards the degradation of the thiazine dye (methylene blue-MB). Approximately 95% of the MB dye was broken down at pH 8 after 150 min of sunlight exposure. The aforementioned results, therefore, suggest that ZnONPs synthesized by implementing environmentally friendly techniques can be employed for a variety of environmental and biomedical applications.

## 1. Introduction

The printing and dyeing industries are among the worst environmental offenders because they release a lot of dyes into wastewater [1]. These dyes are not only unattractive but also dangerous to the environment and living things when present in rivers [2]. The textile industry is now required to remove dyes from its released wastewater due to increasingly stringent environmental regulations. Controlling dye effluent output, however, has proven difficult over time. According to reports, over 7 × 10^5^ tons of dyes are produced annually for synthetic items, with a global yearly output of around 80 million tons [3]. These dyes seriously damage the environment, even when only trace amounts are emitted. Methylene blue is the synthetic dye most frequently used in the textile industry to color textiles [4,5]. Because of the strength of the MB dye molecules, they are difficult for a standard wastewater treatment procedure to break down [6]. MB dye also poisons serotonin and the central nervous system severely [7]. Multiple pathways may be responsible for photocatalyst deactivation, according to the literature that is currently available. In order to change the surface characteristics of the photocatalysts, the aqueous substances may first bind to them through covalent bonds, electrostatic effects, interaction, and secondary bonds. Second, the altered surface charge may cause the photocatalysts to aggregate, and a strong adsorbate–surface contact may cause the photocatalysts to dissolve [8]. Numerous studies have shown that exposure to dye-contaminated water can cause damage to various organs, including the thyroid, conjunctiva/cornea, brain, kidney, liver, gastrointestinal tract, and reproductive system [9,10,11]. Therefore, in today’s dye wastewater treatment, effective dye removal is a hotly debated topic. The contamination brought on by dye effluent has increased interest in its biological, chemical, and physical remediation. The most popular techniques for sequestering and decolorizing dyes are adsorption, coagulation–flocculation, ion exchange, catalytic degradation, chemical precipitation, and ion exchange [2,12,13]. In order to reduce dangerous pollutants in indoor air, many techniques, such as biological degradation, thermal and non-thermal plasma treatment, membrane separation, and photocatalytic oxidation (PCO), have been investigated to date. However, photocatalytic oxidation still has drawbacks, such as quick catalyst deactivation, lower effectiveness under typical working conditions, and the formation of undesirable byproducts [14]. However, these traditional techniques offer some disadvantages, such as limited efficiency, high costs, and unintended side effects [15]. Materials used in photocatalytic activity ought to be safe and resistant to photo-oxidative degradation. Nanotechnology is concerned with creating materials at the nanoscale, which ranges from 1 to 100 nm. In comparison to bulk, the material’s nanoscale size dimension displayed optimal physicochemical characteristics [16,17]. Nanomaterials have generated a lot of attention because of the many applications they have in fields as diverse as medicine, biotechnology, the food industry, agriculture, transportation, national security, sensors, packaging, information technology, aerospace, textiles, and cosmetics [18,19,20,21,22,23,24,25]. Due to their successful applications in key disciplines such as catalysis, advanced nanomaterials have gained more researchers’ interest recently [26,27]. Adsorption, however, is a dependable dye removal method that is operationally easier, economically feasible, highly effective, and has a strong regeneration capacity [28]. Additionally, this technique is renowned for being simple, flexible, and easy to utilize. Infectious illnesses, which cause a large number of deaths globally, are another big issue facing the medical industry [29]. Many dangerous microbes that cause numerous illnesses have been detected in dying wastewater. In addition, improper use of antibiotics and a lack of scientific resources to create such medications have resulted in mutations and generations of microorganisms that are resistant to them [30]. In order to prevent the spread of diseases and give a fresh perspective on therapy, it was important to look for novel therapeutic approaches, such as the use of nanotechnology. At low concentration levels (ppb–ppm), conventional semiconductor nanoparticles frequently exhibit poor pollutant adsorption capacities [14]. As an important micronutrient, zinc (Zn) is a key component of all six types of enzymes, which include oxidoreductases, lyases, isomerases, transferases, hydrolases, and ligases. It also plays a key role in many essential metabolic processes, including the manufacture of photosynthetic pigments [31]. Zinc protects membranes from oxidative and peroxidative damage by maintaining membrane integrity and stabilizing permeability. One of the most significant metal oxides, ZnO nanoparticles, have unique therapeutic effects as well as fungicidal, antibacterial, and catalytic capabilities. ZnONPs may be produced using a variety of methods, such as hydrothermal, solvothermal, chemical, sonication, precipitation, microwave, etc., but in modern times, a biological synthesis of ZnONPs using plant-derived products is widely employed [32,33,34]. ZnO nanoparticles are more effective at blocking UV radiation than bulk ZnO due to their high surface area-to-volume ratio [33]. There are several metabolites present in fungal biomass extracts that easily convert the precursor molecule to zinc ions and then to ZnONPs [35]. *Pluchea indica* Less., a member of the Asteraceae family, is a dual-purpose plant that is mostly found in tropical and subtropical areas. According to chemical analysis results, flavonoids, thiophenes, quinic acids, and other phenolic acids are this plant’s primary ingredients [36]. The most prevalent dietary polyphenolic chemicals among them are quinic acids, which are abundantly present in tea, coffee, and other foods. Numerous bioactivities, including anti-inflammatory and hepatoprotection, have been documented [37]. Furthermore, *P. indica’s* high concentration of phenolic chemicals has led to research into and certification of its free radical scavenging capacity, which revealed that it has an antioxidation impact [38]. Herein, this study aims to biosynthesize ZnONPs using *P. indica* leaf extract for the first time, which is easy to use, ecofriendly, and safe. Moreover, the study aims to characterize biosynthesized ZnONPs by numerous techniques. Finally, to assess the antimicrobial activity and photocatalytic degradation.

## 2. Results and Discussion

### 2.1. Biosynthesis of ZnONPs Using Leaf Extract of P. indica

Since they are inexpensive and require minimal maintenance, plants are regarded as nature’s chemical factories. Many plant parts, including fruit, leaves, stems, and roots, have frequently been used for the green production of nanoparticles because of the large phytochemicals they produce [39]. Iron, gold, silver, zinc oxide, and other nanoparticles have all been created simply using environmentally friendly methods [40]. Metallic ion bioreduction is caused by phytocompounds such as polyols, terpenoids, and polyphenols that are present in plant extracts [41]. As a result, efforts to synthesize ZnONPs using various techniques have intensified. In the current study, ZnONPs were made using a green process that is quick, easy, environmentally friendly, and economically feasible. Zinc ions were reduced, capped, and stabilized using leaf extracts of *P. indica*. to reduce the drawbacks associated with chemical and physical procedures, green approaches (plants, fungi, bacteria, actinomycetes, and yeasts) are chosen for the manufacture of metal and metal oxide NPs [42]. In this instance, the biocatalyst for reducing zinc ions to generate ZnONPs was the leaf extract of *P. indica*. The color change of the extract from pale green to turbid white after mixing with Zn (CH_3_COO)_2_.2H_2_O indicates the formation of ZnONPs. This color shift is a result of the NPs’ surface plasmon resonance becoming excited [43]. According to Fouda et al. [43], the formation of white ZnONPs was achieved by mixing the aqueous extract of *U. fasciata* with the zinc acetate solution. On the other hand, the biosynthesis of ZnONPs was performed by using *Delphinium uncinatum,* which was utilized for its anti-aging, cytotoxic, antibacterial, anti-diabetic, and anti-inflammatory properties. Llashin et al. [44] used *Ziziphus spina-christi* for the green biosynthesis of ZnONPs and selenium nanoparticles. 

### 2.2. Characterization of ZnONPs 

**Figure 1:**
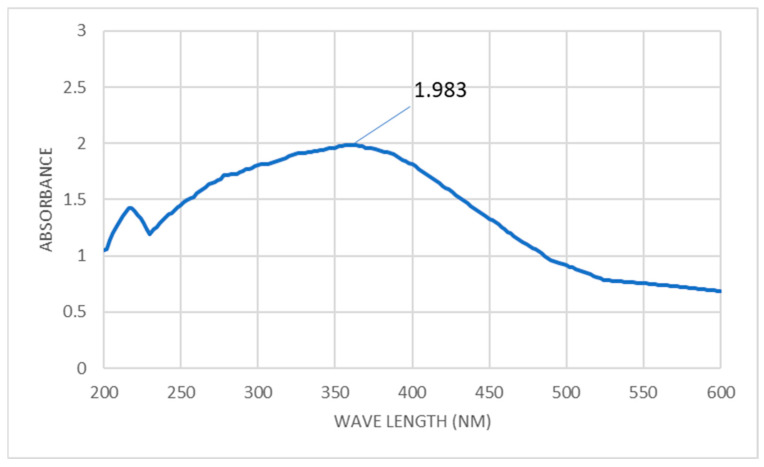
UV–vis spectra of biosynthesized ZnONPs at wavelength 200–800 nm.

The precipitate’s hue shifting to white indicates the presence of ZnONP. In order to find the maximal surface plasmon resonance, the absorbance of the generated color was measured in the 200–600 nm range. After 24 h, the greatest SPR for biosynthesized ZnONPs was recorded at 360 nm (Figure 1). According to Majhi and Kuiri [45], the metal-dielectric constant, the size, and form of the metal NPs, the surrounding medium, and the frequency of the SPR are all variables that affect its width. Earlier studies on the green manufacturing of ZnONPs revealed peak values between 320 and 380 nm [46]. The UV-visible absorption spectra of the biosynthesized ZnONPs from *Vernonia cinerea* leaf extract are 360 nm, which is the same result as that reported by Azim et al. [47]. Kumar et al. [48] found an absorbance peak at the resonance wavelength of 270 nm, which confirmed the existence of ZnONPs in the aqueous solution. According to Vijayakumar et al. [49], the UV spectra of Ae-ZnONPs include two peaks located at 275 and 380 nm, respectively. According to Shubha et al. [50], hexagonal ZnO NPs had an absorption maximum of 368 nm. Additionally, the zinc acetate was successfully transformed into the end product (ZnONPs), as evidenced by the maximum SPR being recorded at 380 nm. [51].

**Figure 2:**
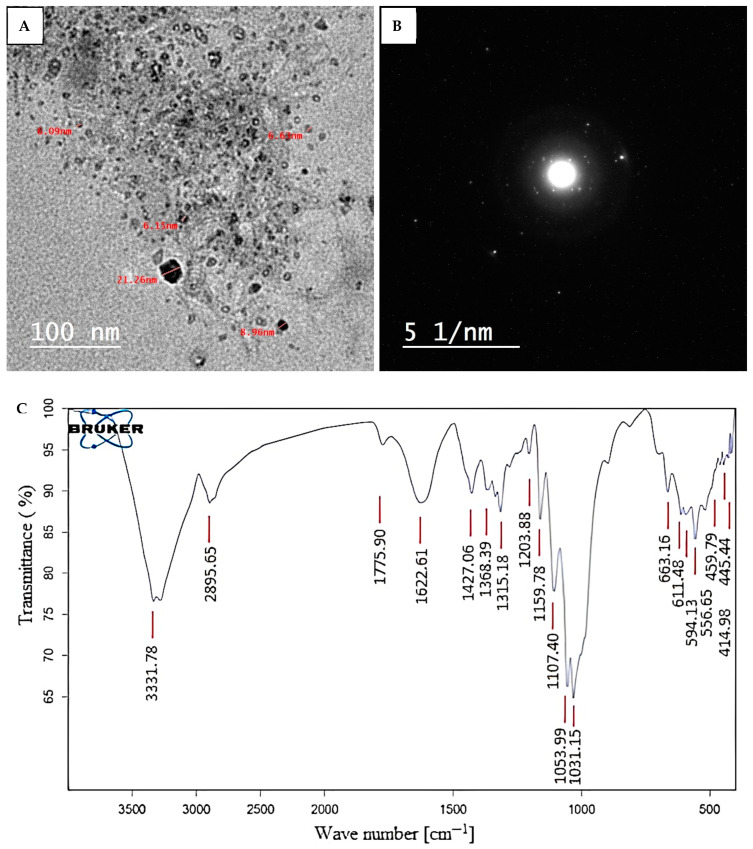
Characterization of biosynthesized ZnONPs. (**A**) TEM image; (**B**) SAED pattern; (**C**) FT-IR spectra.

FT-IR analysis was used to determine the different functional groups present in the cell-free filtrate and their functions in creating and stabilizing ZnONPs. As seen in Figure 2, the FT-IR chart exhibits peaks at certain wave numbers, 3331, 2895, 1775, 1622, 1427, 1368, 1315, 1203, 1159, 1107, 1053, 1031, 663, 611, 594, 556, 459, 445, and 414 cm^−1^. The wide peak at 3331 cm^−1^ indicates the presence of the -OH group [52], whereas the observed peak at 2895 cm^−1^ represents the aldehyde group (CHO) [48]. Conversely, the peak bands at 1622, 1427, 1368, 1315, 1203, 1159, and 1107 cm^−1^ are related to the current carbonyl group (C=O), C-N bonds in aromatic stretching, and C-O [53,54,55]. The peaks of the NPs were variable during biosynthesis. At peaks of 400 to 600 cm^−1^, as previously reported [50,56,57], the successful synthesis of ZnONPs was verified. Final confirmation of the zinc oxide bond comes from the band seen at 414 cm^−1^. The information gathered confirmed the occurrence of a variety of functional groups, such as alkanes, alkenes, aliphatic, aromatic amines, and alkyls, which are present in the leaf extract of *P. indica* and are crucial for the stabilization, capping, and reduction of ZnONPs. Similar results were obtained by Dias et al. [58], who found that FT-IR analysis revealed a strong peak at 432.05 cm^−1^, showing the characteristic Zn-O bond. For the zinc oxide nanoparticles produced using a green approach, Al-Dhabi and Arasu [59] achieved a peak of 417 cm^−1^. Rajivgandhi et al. [60] have identified the peaks for the ZnO nanoparticle in the 400–4000 cm^−1^ wavelength region that was made using *Streptomyces enisocaesilis*.

The investigation of the morphological properties of synthesized NPs, such as their size, aggregation, and shape, may be performed with the use of transmission electron microscopy (TEM). As shown, phytochemicals produced by P. indica can reduce or cap zinc acetate and create spherical, widely scattered ZnONPs (Figure 2A,B). The average diameter of biosynthesized ZnONPs was 12.0 ± 2.2 nm, with sizes ranging from 6 to 21 nm. Figure 2B displayed the ZnONPs’ area selected electron-diffraction (SAED) patterns, which exhibited good sharp rings and demonstrated the crystal structure of the ZnONPs. Spherical ZnONPs were successfully generated using the marine macroalgae Ulva fasciata Delile, with an average size of 10.62 nm and a size range of 3–33 nm [61]. Additionally, Abdo et al. [51] created spherical ZnONPs with diameters ranging from 6 nm to 21 nm. Numerous factors, including surface features, size, coating or capping agent, shape, reactivity, and solubility, can affect the activity of NPs [62]. Additionally, Aziz et al.’s [63] production of zinc oxide nanoparticles with a spherical shape and a size range of 28 to 42 nm was successful. However, Rajivgandhi et al.’s [60] synthesis of ZnONPs showed the diameter of the nanoparticle varying between 199 and 326 nm for the HP05 sample and between 12 and 35 nm for the HP01 sample. These findings lead us to assume that the synthesized ZnONPs used in the current work would have high activity due to their reduced sizes.

**Figure 3:**
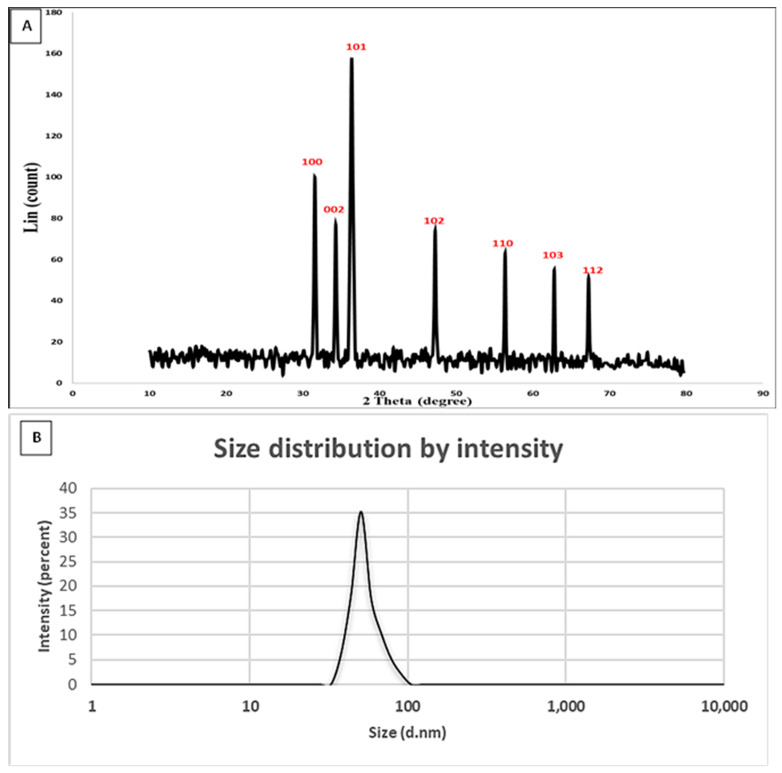
(**A**) XRD analysis, (**B**) DLS analysis of biosynthesized ZnONPs.

Particle size and size distribution investigations may be performed using a variety of approaches. Dynamic light scattering (DLS) is one such well-liked method for figuring out the particle size and size distribution of nanoparticles. The average particle size, distribution, and polydispersity index are calculated by Brownian motion by measuring the light scattering. The analysis revealed that the average particle size of the biosynthesized zinc oxide nanoparticles was 50.7 nm, and their PDI was 0.31. Figure 3B displays the size distribution of intensity. Similarly, the particle size analysis was performed for zinc oxide nanoparticles, which are synthesized by *Cordyceps militaris* [58]. According to Mohamed et al. [52], the obtained ZnONPs were poly-dispersed mixtures with average diameters of 135.5 nm (86.4%) and 163.34 nm (92.7%) for nanorod and hexagonal ZnONPs, respectively.

By using XRD, the crystallinity of biosynthesized ZnONPs in the range of two theta values was examined (Figure 3A). The spectra show the existence of seven prominent peaks for Bragg reflection at (100), (002), (101), (102), (110), (103), and (112) at two theta values of 31.6°, 34.3°, 36.2°, 47.2°, 56.4°, 62.8°, and 67.2°, respectively. According to the Diffraction Standards JCPDS data (Zincite, JCPDS 5-0664) for polycrystalline wurtzite structure, the collected results demonstrated the crystallinity of ZnONPs [64]. The XRD pattern for the crystalline nature of biologically produced ZnONPs was consistent with our observations [51,52]. The particle size of the created NPs was determined using the Debye-Scherrer equation. The average ZnONP size that could be analyzed by TEM in this instance was 21.9 nm, and the FWHM (2ϴ) value was 0.3981. The effective manufacturing of small ZnONPs of the previously reported sizes is indicated by the widening of the bases of Bragg’s diffraction peaks [58,64]. The different peaks recently approximated the diffraction planes (100), (260), (002), (101), (102), and (101). The findings support the biofabricated ZnONPs’ wurtzite structure [47]. 

**Figure 4:**
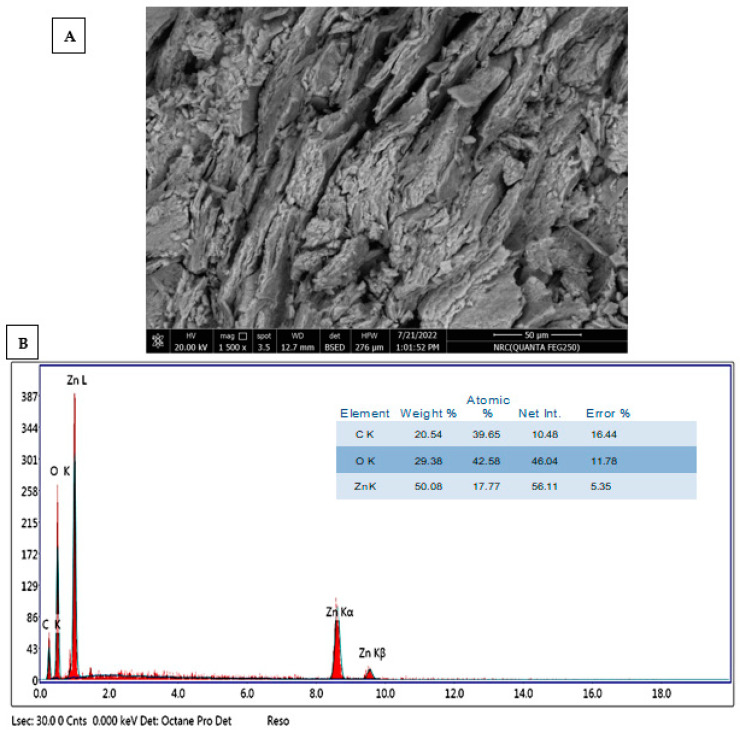
(**A**) Scanning Electron Microscope (SEM) of ZnONPs; (**B**) EDX spectrum showed elemental compositions of biosynthesized ZnONPs.

The surface appearance and qualitative and quantitative elemental compositions of ZnONPs produced by *P. indica* leaf extract were examined using SEM in conjunction with EDX equipment. As shown, the produced ZnONPs had a spherical form and were evenly spread (Figure 4A). The synthesis of ZnONPs was successful, as seen by the prominent peak in the zinc area of the EDX chart (Figure 4B). According to the EDX chart, Zn makes up a large portion of the synthesized components. Figure 4B demonstrates the presence of C, O, and Zn, with weight percentages of 20.5, 29.3, and 50%, respectively. The ZnO nanoparticle’s elemental composition showed that it included 61.57% oxygen and 38.43% zinc, respectively, according to Rajivgandhi et al. [60]. In a manner similar to this, ZnONPs were produced using Zingiber officinale root extract and then analyzed using EDAX. Additionally, elemental analysis of nanoparticles generated from root extract showed that they included around 80% zinc and 19% oxygen [65]. Furthermore, *S. marginatum* and *U. lactuca* produced ZnONPs that were mostly composed of Zn and O with weight percentages of (51.6 and 48.4%) and (48.3 and 51.7%), respectively [61]. Similar to this, Mohamed et al. [66] discovered that Zn (58.3%) and O (20%) were the two main peaks of the EDX spectra for ZnONPs generated by *P. chrysogenum*, in addition to the occurrence of other peaks linked to biomolecules in *P. chrysogenum* filtrate that conjugated with ZnONPs. 

### 2.3. Antimicrobial Activity

Zinc oxide nanoparticles have piqued the interest of researchers and scientists during the last decade due to their numerous potential applications in biomedicine, the environment, and electronics. ZnO nanoparticles are of tremendous interest because of their low cost, safety, and ease of manufacture. In the current study, the biosynthesized ZnONPs using leaf extract of *P. indica* were assessed as antimicrobial agents against Gram-positive and Gram-negative bacteria as well as unicellular fungi, as shown in Table 1. Results displayed that the biosynthesized ZnONPs exhibited antibacterial activity where inhibition zones were 21.83 ± 0.76, 13.0 ± 1.1, 14.9 ± 0.85, 24.26 ± 1.1, and 17.0 ± 1.0 mm toward *E. coli, P. aeruginosa, E. faecalis, B. subtilis* and *S. aureus* respectively, at a concentration of 1000 µg/mL. Moreover, *B. subtilis* was the most sensitive among tested bacterial strains to ZnONPs, where the MIC was 62.5 µg/mL, while *P. aeruginosa* was the least sensitive, where the MIC was 500 µg/mL. Compared to standard antibiotic (SAM), SAM showed weak antibacterial activity against all tested bacterial strains, where MICs were in the range of 500–1000 µg/mL. Furthermore, the biosynthesized ZnONPs revealed potential antifungal activity against *C. albicans* and *C. neoformans* where inhibition zones at concentrations of 1000 µg/mL were 20.67 ± 0.57 and 19.0 ± 1.0 mm, respectively. Moreover, the MIC was 125 µg/mL for both *C. albicans* and *C. neoformans*. Although no previous studies on the biosynthesis of ZnONPs by *P. indica* leaf extract, previous studies reported that leaf plant extracts were used for the biosynthesis of ZnONPs [67,68]. Naseer, Aslam, Khalid, and Chen [67] succeeded in the biosynthesis of ZnONPs using leaf extracts of *Cassia fistula* and *Melia azadarach*, where these nanoparticles showed promising antibacterial activity against *E. coli* and *S. aureus.* Moreover, [69] reported that ZnONPs synthesized by leaf extracts of *Passiflora caerulea* revealed antimicrobial activity toward urinary tract infection-causing microbes. Moreover, Gharpure et al. [70] illustrated that leaf extract of *Neolamarckia cadamba* can be used for the green biosynthesis of ZnONPs and also found that these nanoparticles have antibacterial activity against *B. subitils, S. aureus, P. aeruginosa,* and *E. coli.* Furthermore, *Pisonia alba* leaf extract was used for ZnONPs biosynthesis and exhibited potential antibacterial activity toward Gram-negative and Gram-positive bacteria [71]. Additionally, Chaudhary et al. [72] succeeded in the biosynthesis of ZnONPs using *Aloe vera* peel extract and found ZnONPs have promising antimicrobial activity against *E. coli* (MTCC-41) and *A. niger* (MTCC-404). Bala et al. [73] reported that the biosynthesized ZnONPs using leaf extracts of *Hibiscus subdariffa* had antibacterial activity toward *S. aureus* and *E. coli*. Moreover, ZnONPs were green biosynthesized using cinnamon and bay leaves and displayed antibacterial and antifungal activity against *S. aureus*, *S. epidermidis, E. coli*, *Klebsiella pneumonia,* and *C. albicans.* Moreover, ZnONPs were biosynthesized through a green and eco-friendly method, where *Allium sativum* and *Zingiber officinale* extracts were used, and the biosynthesized ZnONPs showed antibacterial activity against *E.coli, P. putida, S. aureus,* and *Streptococcus pyogenes* [74]. The production of reactive oxygen species (ROS), such as hydroxyl radicals (OH), hydrogen peroxide (H_2_O_2_), and peroxide (O_2_), may be the cause of the antibacterial activity of biosynthesized ZnONPs. A number of processes have been linked to ROS, including the internalization of NPs as a result of proton motive force loss, cell wall disintegration brought on by ZnO-localized contacts, enhanced membrane permeability, and the consumption of dangerous dissolved zinc ions. These have led to oxidative stress-related gene expression, intracellular outflow, and mitochondrial malfunction, which have suppressed cell development and led to cell death [75]. Dwivedi et al. [76] conducted research on the mechanism of ZnO-NPs’ antibacterial action, which they determined to be ROS production as a result of treatment with DCFH-DA dye. When the dye enters the bacterial cell passively, cell esterases break it down, releasing DCFH. Reactive oxygen species (ROS) enable the oxidation of DCFH to DCF, a highly fluorescent chemical known as dichlorofluorescein. The amount of reactive oxygen species (ROS) is correlated with the intensity of the fluorescent signal, which was quantified using flow cytometry or a microplate reader [77].

### 2.4. Photocatalytic Degradation of Methylene Blue-MB Using ZnONPs

**Figure 5:**
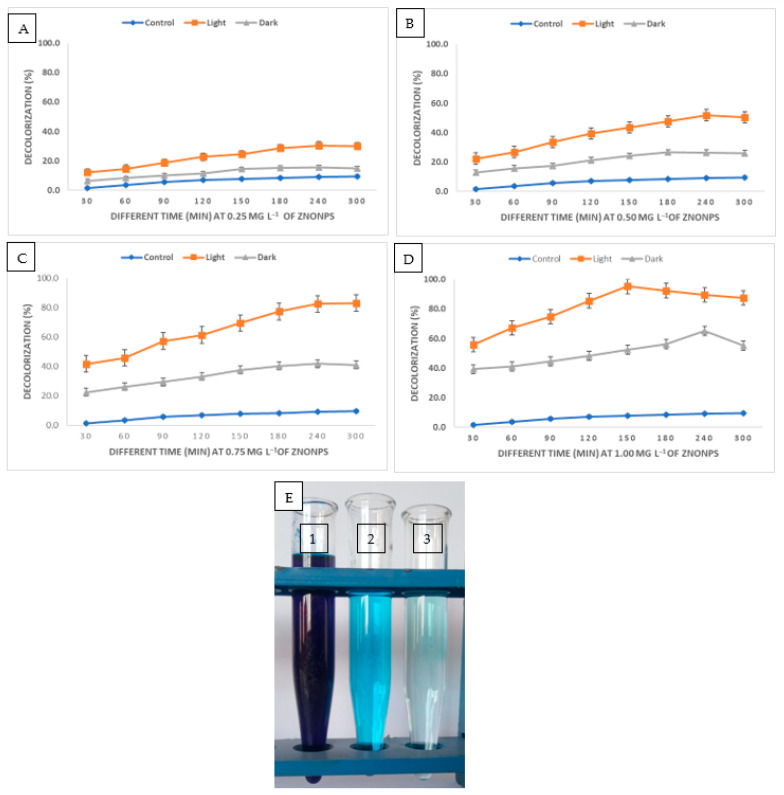
Decolorization percentages of MB dye under various stimulation settings (dark and sunlight), at various ZnONPs concentrations, and over various contact periods (**A**–**D**). Dye decolorization in dark and light environments (**E**) (1–3), where E1 represented the dye control, E2 represented the dye in the dark environment, and E3 represented the dye in the light environment.

Either directly applying high-energy light sources to the surface of the nanomaterials or utilizing a photosensitization approach is used in the degradation process. Nanoparticles are irradiated with light, and direct photocatalytic degradation happens when electrons are moved from the valence band (filled) to the conduction band by the use of light energy. This process is known as photo-excitation [78]. In order to undertake a comparative analysis, the potential of ZnONPs for the decolorization of methylene blue dye was examined in this work at various ZnONPs concentrations (25, 50, 75, and 100 mg) for various contact durations (30, 60, 90, 120, 150, 180, 240, and 300 min) in both light and dark. The data analysis showed the dosage and time dependence of ZnONPs’ catalytic activity. Surprisingly, exposure to light accelerated the biodegradation of ZnONPs more than exposure to darkness (Figure 5A–D). In comparison to the control, which had a decolorization percentage of 9.1 ± 0.35% after 240 min. The decolorization percentages at 0.25 mg mL^−1^ the concentration of ZnONPs reached up to 30.5 ± 0.51% and 15.7 ± 0.72% under sunlight and dark conditions, respectively. At 0.5 mg mL^−1^ of ZnONPs, the decolorization percentages under sunlight stimulation increased to 51.8 ± 1.04% after 240 min. After 180 min, the percentages of decolorization at 0.75 mg mL^−1^ of ZnONPs under light and dark conditions were 77.2 ± 0.34% and 40.3 ± 0.84%, respectively. In the presence of sunlight, the maximum decolorization was attained at 1.0 mg mL^−1^ of ZnONPs with percentages of 95.3± 0.65% after 150 min; while in the absence of sunlight at the same NPs concentration, the decolorization was 56.3 ± 0.93% after 180 min. These findings showed that 1.0 mg mL^−1^ of ZnONPs after 150 min of contact time was the most suitable condition. Saied et al. [79] found that the presence of light stimulators is necessary for the biosynthesized Hem-NPs to effectively degrade CV dye. An increase in ZnONPs concentration results in the greatest dye decolorization because there are more adsorption sites on the NPs’ surface [80]. When compared to complicated solutions made up of many dye types or unidentified compounds, the time needed to decolorize and degrade either pure or one dye was the same [81]. The amount of dye that degrades is reducing as dye molecules compete for binding to the few available reaction sites on the nanoparticles at a greater concentration [80]. The number of active sites on the surface of the zinc oxide nanoparticles reduces as the amount of adsorbent increases, which lowers the adsorbent’s capacity for the adsorption of dye molecules on its surface [82]. The photocatalytic efficiency of ZnONPs revealed higher eco-bioremediation capacities by degrading methylene blue (88.93%) and crystal violet (80.69%) dyes, according to Omran [83]. Additionally, Nguyen et al. [84] showed that the maximal Congo red dye degradation efficiency was 94.85% at 5.0 mg L^−1^ of ZnFe2O4@ZnO nanocomposites concentration and 0.33 g L^−1^ of ZnO dosage. Additionally, after 75 min of exposure to sunlight, almost 80% of the MB dye began to break down at pH 8 [85]. The mycosynthesized ZnO nanoparticles showed potential dye degradation efficiency of up to 90% of fast green dye under photo illumination [48]. 

**Figure 6:**
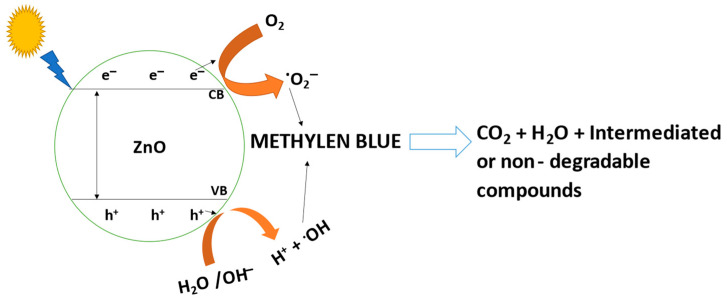
Flowchart showing the mechanism for decolorization and degradation of methylene blue dye by ZnONPs.

One of the most crucial pieces of knowledge to have while creating a system is the mechanism of adsorption. The major mechanism at play between the adsorbent and the adsorbed dye was electrostatic contact. In the adsorption process between the surfaces of the ZnONPs and dye, hydrogen bonds, hydrophobic interactions, and interactions of the ZnO-NPs with the aromatic rings of the dye all played important roles. Additionally, the C=O, OH, NH, and phenyl groups of the ZnONPs, which served as adsorption sites, interacted with the aromatic rings of dye molecules [86]. Zinc oxide is essentially insoluble in aqueous solutions and functions as a flimsy base. ZnONPs’ valence band electrons are photoexcited into their conduction band by sunlight. O_2_ was converted into O_2_^•−^ radicals, which were subsequently converted into hydroxyl radicals as a result of the separation of charges brought on by the electrons in the conduction band. Additionally, the generated hole (h^+^) has a propensity to split water molecules into hydrogen (H^+^) ions and hydroxyl radicals (OH^•^) (Figure 6). As a potent oxidizing agent, the generated hydroxyl radical (OH^•^) is extremely reactive and may be used to degrade dyes and other contaminants [87,88,89].

(1) $$\mathrm{ZnO}\;\frac{\mathrm{Excitation}}{\mathrm{Sunlight}}\;h^{+}+e^{–}$$

h^+^ + H_2_O→H^+^ + ^•^OH (2)

e^–^ + O_2_→^•^O_2_ ^–^ (3)

O_2_ ^−^ + h^+^→ ^•^OH (4)

methylene blue + ^•^OH→CO_2_ + H_2_O + non − degradable products (5)

### 2.5. Reusability of ZnO Nanoparticles as Catalyst

**Figure 7:**
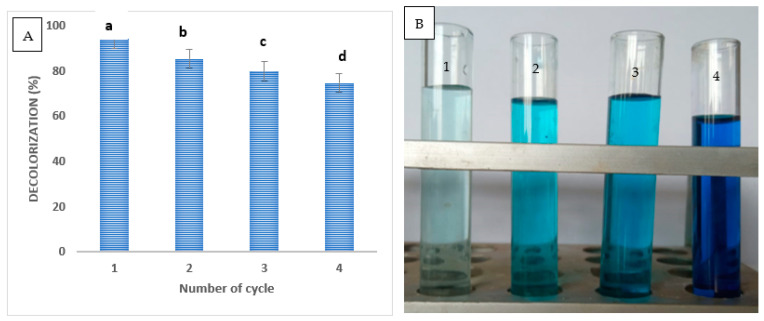
(**A**) ZnONPs recycling in methylene blue removal process; (**B**) At cycles 1–4 of the methylene blue removal cycle, the color changes. Letters a, b, c and d mean significance power.

In this work, the stability of biosynthesized ZnONPs for reuse in dye wastewater treatment was investigated. In this study, the regeneration of the employed ZnO nanoadsorbents from the dye solutions was carried out to ensure the appropriateness of our systems from the standpoint of industrial applications. ZnONPs were washed with a suitable proportion of water and ethanol for the concerned purpose after being centrifuged out of the dye solutions that had been treated. To make powdered ZnONPs, the sediment pellets were dried in an oven. In a similar way, ZnONPs were tested for reusability. Even after the fourth cycle of reusability, the dye removal percentage using ZnONPs showed a removal of 74.7% (Figure 7A,B). After four reuses, the dye removal efficiency was 87%, according to Rasool et al. [80]. These findings are consistent with other studies [87,90,91]. The unavoidable decrease in catalyst performance was mostly caused by the reduction of the catalytic site, the concentration of metal leaching, and the adsorption of intermediate products on the catalytic site [79].

### 2.6. Comparison between ZnO and Other Solid Adsorbents and Photocatalysts

A comparison of the photocatalytic activities for ZnO and other published nanomaterials is presented in Table 2 [92,93,94,95,96,97]. Based on the tabulated data for photocatalytic degradation of methylene blue, we can conclude that nano zinc oxide and Other solid adsorbents are used in environmental applications.

## 3. Materials and Methods

### 3.1. Materials

Zinc acetate dihydrate (Zn (CH_3_COO)_2_·2H_2_O), methylene blue, and sodium hydroxide (NaOH) were analytical-grade compounds that were purchased from Sigma-Aldrich for use in this research. 

### 3.2. Preparation of P. indica Leaf Extract

*Pluchea indica* leaves were obtained from Egypt’s Giza Governorate. The recovered leaves were thoroughly cleansed with double-distilled water and left to dry for 5 days to eliminate any contaminants. Next, 5 g of the chopped material was combined with 100 mL of deionized water to create the extract, which was then heated at 65 °C for 60 min before being decanted. For use within a week, the supernatant was centrifuged for 10 min at 10,000 rpm and stored at 4 °C [98].

### 3.3. Biosynthesis of ZnONPs Using Leaf Extracts of P. indica

**Figure 8:**
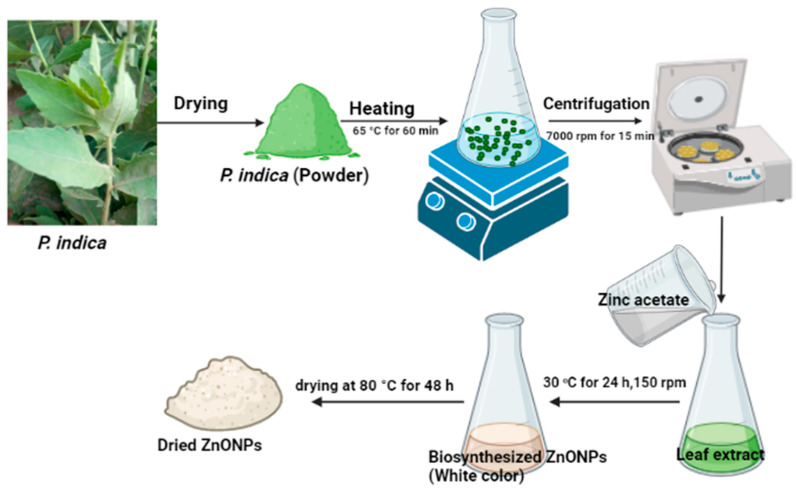
Preparation of *P. indica* leaf extract and biosynthesis of ZnONPs.

To biosynthesize ZnONPs, zinc acetate (3 mM) was added to P. indica leaf extract and agitated at 150 rpm for 24 h at 30 °C. At the end of the incubation period, a white tint was seen as a result of ZnONPs production. Drying the ZnONPs took 48 h at 80 °C. The ZnONPs product was eventually gathered and put through additional testing (Figure 8).

### 3.4. Characterization of ZnONPs 

The characterization of ZnONPs was performed using Ultraviolet-visible (UV-vis) spectra, and the development of ZnONPs was tracked by observing changes in the color of the solution. UV-vis spectra were used to monitor the biosynthesis of ZnONPs in a colloid solution because surface plasmon stimulation causes it to produce a strong absorption peak. At wavelengths between 200 and 600 nm, the JENWAY 6305 Spectrophotometer was used to observe color change. The size and shape of nanoparticles were determined using a TEM (JEM-1230, Japan, Akishima, Tokyo 196-8558) and selected area electron diffraction (SAED). Using a Malvern Zetazier Instrument (Malvern, UK), DLS measurements were used to assess the particle size distribution of ZnONPs. The energy dispersive spectroscopy (EDX) apparatus (JEOL, JSM-6360LA, Tokyo, Japan) was coupled to a scanning electron microscope (SEM), which was used to examine the elemental composition of mycosynthesized ZnONPs. The potential biomolecules included in biosynthesized ZnONPs were identified using FTIR. The material was scanned using a Fourier Transform-Infrared Spectrometer (Agilent System Cary 660 FTIR model) in the infrared region of 400–4000 cm^−1^. X-ray diffraction patterns were analyzed using the X’Pert Pro X-ray diffractometer (Philips, Eindhoven, Netherlands). The average crystallite size of ZnONPs may also be calculated using the Debye–Scherrer equation. ZnONPs’ crystalline structure was identified in the 2ϴ range of 10° to 80°.

### 3.5. Antimicrobial Activity

Biosynthesized ZnONPs were evaluated for antimicrobial activity toward Gram-negative bacteria (*Escherichia coli* ATCC 25922 and *Pseudomonas aeruginosa* ATCC 27853), Gram-positive bacteria (*Enterococcus faecalis* ATCC 29212, *Staphylococcus aureus* ATCC 25923 and *Bacillus subtilis* ATCC 6051) and unicellular fungi (*Candida albicans* ATCC 90028 and *Cryptococcus neoformans* ATCC 14116). Minor modifications were made to the Clinical Laboratory Standard Institute’s guideline M51-A2 [99] when performing the diffusion in agar test. Individually, 100 µL of ZnONPs, leaf extract, standard antibiotic (Ampicillin/sulbactam), reference antifungal (Nystatin), and zinc acetate at a concentration of 1000 µg/mL were added to the agar well, and then plates were put in the refrigerator for 2 h followed by incubation at (37 °C for 24 h)/(30 °C for 48 h) for bacterial/fungal strains, respectively. Then, inhibition-zone diameters were measured. ZnONPs and AMC/FLU were prepared at a variety of concentrations ranging from 1000 to 3.9 g/mL, and their respective MICs were then tested against a number of bacterial and fungal strains [100,101].

### 3.6. Photocatalytic Activity 

The catalytic activity of biosynthesized ZnONPs was examined using methylene blue under both dark and light stimulation. The experiment involved mixing 50 mL of MB solution with various amounts of ZnONPs (25, 50, 75, and 100 mg) over a range of contact periods (30, 60, 90, 120, 150, 180, 240, and 300 min). A 250-watt halogen lamp served as the illumination source. Briefly, 50 mL of the MB solution was added to a specified ZnONPs concentration after the MB solution had been made at a concentration of 10 mg L^−1^. Earlier in the experiment, the prior mixture was agitated for 30 min to reach the balance of absorption and desorption. The mixture was then exposed to light and incubated at room temperature with aeration. For comparison analysis, the same experiment was carried out once again under identical circumstances under dark irradiation. The optical density of the clear supernatant was measured using spectrophotometers, M-ETCAL, OK International Ltd., Eastleigh, UK, at λ max of MB (663 nm), after 2.0 mL of each treatment had been centrifuged at 5000 rpm for 10 min [102]. The following calculation was used to compute the decolorization percentages of MB color removal:

D% = ((dye 1 − dye 2)/dye 1)) × 100

where D% represents the ratio of decolorization, dye (1) represents starting absorbance, and dye (2) represents final absorbance. For the fourth cycle, the catalyst’s reusability in the degradation of MB was accomplished under ideal circumstances. The catalyst from the first cycle was recovered by centrifugation, subjected to two washings with distilled water, and then oven-dried at 80 °C to minimize water content before being used in the second cycle.

### 3.7. Statistical Analysis

All results presented in this study are the mean of three independent replicates. The SPSS v17 statistical software was used to analyze variance in the data. The mean difference between the treatments was analyzed by the Tukey HSD test at a significant level of *p* ≤ 0.05.

## 4. Conclusions

In this study, *P. indica* leaf extract was used, for the first time, to synthesize ZnONPs utilizing a simple, effective, and eco-friendly method. The size, shape, and structure of the biosynthesized NPs were evaluated using transmission electron microscopy, Fourier transform-infrared spectroscopy, X-ray diffractometry, and dynamic light scattering investigations. Broad XRD peaks were present in the biosynthesized ZnONPs, confirming their nanocrystallinity and average size of 21.9 nm. The capping activity on the surface of ZnONPs is supported by the presence of polyphenols’ stretching vibrations, which are present in the extract. The ZnONPs were observed at 360 nm. The biosynthesized ZnONPs exhibited antibacterial and antifungal activity against Gram-positive, Gram-negative, and unicellular fungi. The ZnONPs were found to have high efficacy in dye removal and to be simple to reuse four times. The highest dye decolorization was 95% after 150 min. Our findings highlight the significance of choosing the right plant to produce a particular NP shape in relation to the characteristics and potential uses of biosynthesized nanoparticles.

## Figures and Tables

**Table 1:** Antimicrobial activity of biosynthesized ZnONPs.

Microbial Strains	*P. indica* Leaf Extract	Zinc Acetate	ZnO NPs		SAM/NS **	
	IZ/mm *	IZ /mm	IZ/mm	MIC	IZ	MIC
*E. coli*	ND	ND	21.83 ± 0.76 ^ab^	125	12.5 ± 0.5 ^b^	500
*P. aeruginosa*	ND	ND	13.0 ± 1.1 ^f^	500	9.33 ± 0.57 ^c^	1000
*E. faecalis*	ND	ND	14.9 ± 0.85 ^ef^	500	12.5 ± 0.86 ^b^	500
*B. subtilis*	ND	ND	24.26 ± 1.1 ^a^	62.5	15.4 ± 0.53 ^a^	125
*S. aureus*	ND	ND	17.0 ± 1.0 ^de^	250	9.73 ± 0.46 ^c^	1000
*C. albicans*	ND	ND	20.67 ± 0.57 ^bc^	125	11.83 ± 0.76 ^b^	500
*C. neoformans*	ND	ND	19.0 ± 1.0 ^cd^	125	10.16 ± 0.29 ^c^	1000

* IZ means inhibition zone, ** SAM/NS means Ampicillin–sulbactam/nystatin. Letters ^a, b, c, …^ mean significance power.

**Table 2:** Photocatalytic degradation of methylene blue dye using ZnONPs and modified ZnONPs.

Type of Nanomaterial	Type of Dye	Photocatalysis	References
In/ZnO nanoparticles	methylene blue	89%	[92]
ZnO/C	methylene blue	95%	[95]
ZnONPs	methylene blue	92.5%	[93]
Ag–ZnO/g-C3N4/GO nanocomposite	methylene blue	93.43%	[94]
N-doped ZnO	methylene blue	95.3%	[97]
Gd-doped ZnO nanoparticles	methylene blue	93%	[96]

## Data Availability

All data and materials are viable.
